# Diagnostic Accuracy of a Loop-Mediated Isothermal PCR Assay for Detection of *Orientia tsutsugamushi* during Acute Scrub Typhus Infection

**DOI:** 10.1371/journal.pntd.0001307

**Published:** 2011-09-13

**Authors:** Daniel H. Paris, Stuart D. Blacksell, Pruksa Nawtaisong, Kemajittra Jenjaroen, Achara Teeraratkul, Wirongrong Chierakul, Vanaporn Wuthiekanun, Pacharee Kantipong, Nicholas P. J. Day

**Affiliations:** 1 Mahidol-Oxford Tropical Medicine Programme, Faculty of Tropical Medicine, Mahidol University, Bangkok, Thailand; 2 Center for Tropical Medicine, Nuffield Department of Clinical Medicine, Churchill Hospital, Headington, Oxford, United Kingdom; 3 Chiang Rai Prachanukhao Hospital, Chiang Rai, Thailand; University of Texas Medical Branch, United States of America

## Abstract

**Background:**

There is an urgent need to develop rapid and accurate point-of-care (POC) technologies for acute scrub typhus diagnosis in low-resource, primary health care settings to guide clinical therapy.

**Methodology/Principal Findings:**

In this study we present the clinical evaluation of loop-mediated isothermal PCR assay (LAMP) in the context of a prospective fever study, including 161 patients from scrub typhus-endemic Chiang Rai, northern Thailand.

A robust reference comparator set comprising following ‘scrub typhus infection criteria’ (STIC) was used: a) positive cell culture isolate and/or b) an admission IgM titer ≥1∶12,800 using the ‘gold standard’ indirect immunofluorescence assay (IFA) and/or c) a 4-fold rising IFA IgM titer and/or d) a positive result in at least two out of three PCR assays.

Compared to the STIC criteria, all PCR assays (including LAMP) demonstrated high specificity ranging from 96–99%, with sensitivities varying from 40% to 56%, similar to the antibody based rapid test, which had a sensitivity of 47% and a specificity of 95%.

**Conclusions/Significance:**

The diagnostic accuracy of the LAMP assay was similar to realtime and nested conventional PCR assays, but superior to the antibody-based rapid test in the early disease course. The combination of DNA- and antibody-based detection methods increased sensitivity with minimal reduction of specificity, and expanded the timeframe of adequate diagnostic coverage throughout the acute phase of scrub typhus.

## Author Summary

There is an urgent need for alternative diagnostic methods for scrub typhus, but evaluation of these is hampered because the current serological gold standard (IFA) is imperfect. In a study from Thailand, 3 of 20 (15%) patients with fever had a positive *Orientia tsutsugamushi* PCR result despite negative serology. These findings could reflect potential benefits of the PCR assay in detecting rickettsaemia before antibody responses set in and/or a diagnostic advantage in endemic areas with high background levels of antibody in the population. Serology is complicated by the heterogeneity of strains present in Southeast Asia, but high resource costs and training make realtime PCR assays impractical for many areas where scrub typhus is endemic. This is where the new LAMP methodology has potential: it is inexpensive, simple to perform and requires only a waterbath or simple heating block instead of a thermocycler. In the context of a prospective fever study in a scrub typhus-endemic area in Thailand, the results support the validity of LAMP methodology for the diagnosis of scrub typhus, highlight the difficulties in comparing antibody- with DNA-based methods and also contribute towards understanding the dynamics of bacteraemia in this under recognised and under studied disease.

## Introduction

Scrub typhus, caused by *Orientia tsutsugamushi*, is an important acute febrile illness in the Asia-Pacific region and is endemic in Thailand and Laos [1]–[2]. The clinical discrimination of scrub typhus from other undifferentiated fevers, such as dengue, malaria and leptospirosis, is often very difficult because the clinical symptoms are similar. Diagnosis of scrub typhus infection has relied on the detection of *O. tsutsugamushi* antibodies during the acute and convalescent phases of the disease and the gold standard serological assay is the indirect immunofluorescence antibody assay (IFA) [3]–[4]. The diagnosis of rickettsial disease at the time of patient admission is difficult. Many diagnostic criteria can be used to provide a diagnosis, including bacterial isolation, dynamic serology, higher admission titers than the average endemic background titers, and DNA-based or antigen-based tests based on detecting bacteraemia in patients with acute disease.

However many of these criteria have limitations in scrub typhus: serology is complicated by the significant observed antigenic heterogeneity of disease-causing strains; positivity cut-off titers for single admission IgM are unreliable for different areas of endemicity and demonstrate wide ranges; dynamic serology requires a follow-up sample and cannot guide patient management, bacterial isolation while very specific is hampered by low sensitivity and requires even more time. Further the ‘diagnostic window’ for antigen- or DNA-based assays is limited and remains to be determined in scrub typhus patients. While these methods allow for a retrospective diagnosis with high confidence, to date no single diagnostic test is capable of reliably detecting all scrub typhus cases on admission. There is an urgent need to develop rapid and accurate point-of-care (POC) technologies for acute scrub typhus diagnosis in low-resource, primary health care settings to guide clinical therapy [5].

In this study we aimed to evaluate the diagnostic accuracy for a loop-mediated isothermal PCR assay (LAMP) in the diagnosis of acute scrub typhus infection, targeting the *groEL* gene, encoding the 60 kDa Heat-Shock-Protein of *Orientia tsutsugamushi* [6]. LAMP methodology is based on isothermal amplification of DNA with high specificity and efficiency [7]–[8]. LAMP employs a robust DNA polymerase and a set of three primer pairs that produce a specific double hairpin DNA template. This product is then amplified and concatenated with very high efficiency, leading to DNA concentrations close to 1 µg/µL within 60 to 90 minutes. Endpoint determination is performed by measuring turbidity, which is caused by the precipitation of magnesium pyrophosphate as a by-product of the reaction [9].

The evaluation of this assay was performed in the context of a prospective fever study in the scrub typhus-endemic environment of Chiang Rai, northern Thailand.

## Materials and Methods

### Ethics statement

Ethical approval for this study was granted by following local ethical committees: Chiang Rai Hospital, the Thai Ministry of Public Health, the National Ethics Committee for Health Research and the Oxford Tropical Research Ethics Committee, UK. All patients provided provided written informed consent prior to sample collection.

### Patient samples

A total of 161 patients were prospectively recruited during a fever study conducted in a scrub typhus endemic area, at Prachanukhao Hospital in Chiang Rai, Northern Thailand. Over one calendar year (08/2007-08//2008) the study recruited in-patients over 15 years old with acute fever of less than 2 weeks duration, no evidence of primary focus of infection, three negative malaria blood smears and who provided written informed consent.

Full blood admission samples in EDTA and serum were collected from patients following written informed consent. Convalescent specimens were tested for serology only (defined as follow-up serum to be paired with admission sera) and were available for 138/161 patients, representing a follow-up rate of 86%. Serum samples were collected and stored at −80°C until testing. For DNA templates, buffy coat samples were collected at the clinical study sites and stored at −80°C until DNA-extraction using the Qiagen Mini Blood kit (Qiagen, USA).

### Indirect immunofluorescence assay

Scrub typhus (*O. tsutsugamushi* pooled Karp, Kato, Gilliam antigens) and murine typhus (*R. typhi* Wilmington strain) IgM antibodies were detected using the gold standard indirect micro immunofluorescence assay (IFA) [4], on IFA slides purchased from the Australian Rickettsial Reference Laboratory (Geelong, Australia). Briefly, patient sera underwent serial 2-fold dilutions from 1∶50 to 1∶25,600 in phosphate-buffered saline containing 2% (w/v) skim milk powder (PBS-SMP) and transferred to the IFA slides. After incubation in a humidified atmosphere for 30 minutes at 37°C and 3 washing cycles in PBS, anti-human IgM FITC conjugate (Jackson, USA), diluted in PBS-SMP diluent containing 0.00125% (w/v) Evans Blue counterstain was applied to all wells and incubated in a humidified atmosphere for 30 minutes at 37°C. All slides were prepared by two trained and experienced laboratory staff members, which were blinded to all patient data. Results were examined by epifluorescence microscopy (Olympus BX50, Japan) at 400x magnification and the endpoint was determined as the highest titer displaying specific fluorescence.

### Scrub typhus immunochromatographic test

The scrub typhus immunochromatographic test (PanBio, Australia) (ST ICT) was performed on 159/161 (99%) admission specimens according to the manufacturers instructions. Briefly, 5 µl of serum was applied to the reagent pad of the ICT strip followed by 2 drops of buffer. Results were read visually exactly 10 minutes later and recorded as positive, equivocal or negative for the presence of anti-*O. tsutsugamushi* IgM antibody. As the tests were performed in a routine hospital laboratory with staff rotation, the ICTs were performed and read individually by trained operators under the direction of the study supervisor at Prachanukhao Hospital, Chiang Rai, Northern Thailand.

### *In vitro* isolation of *O. tsutsugamushi*

*In vitro* isolation of *O. tsutsugamushi* was performed at a biocontainment level 3 laboratory in Bangkok using a previously described method [10]. Blood samples were centrifuged at 300xg for 10 minutes, 1 ml of Buffy coat collected by aspiration and mixed with 1 ml of growth medium (RPMI 1640 medium containing 10 mM HEPES (PAA, Austria) supplemented with 10% (v/v) fetal calf serum (PAA, Austria). The sample was then inoculated into two 25 cm2 tissue culture flasks containing 90% confluent Vero cell monolayers (ATCC CCL81). The flasks were centrifuged for 30 min at 500xg at ambient temperature (25°C), incubated at 37°C for 60 min, the inoculum removed, the monolayer washed twice and 10 ml of fresh growth medium added and incubated in a 5% CO2 environment at 37°C. The presence or absence of *O. tsutsugamushi* was ascertained by IFA every 7 days.

### PCR assays

#### Nested 56 kDa PCR assay

A previously described nested conventional PCR assay targeting the 56 kDa gene was performed, with reduction of the reaction end volume from 100 µL to 25 µL [11].

#### 47 kDa based real-time PCR assay

This assay used previously published primers [12] with endpoint visualisation by intercalating SYBR green as previously described [13].

#### *GroEL*-based real-time PCR assay

Recently we designed a highly sensitive real-time PCR primer set based on the *groEL* gene with a specific product of 160 bp length and melt temperature of 84.5°C. A serial dilution of plasmids served as external controls for quantitation assays and melt curve analysis was performed for product differentiation [6].

#### LAMP Assay

All LAMP assay reactions were performed in triplicate, each in separate runs, using the methods previously described [14]. A consensus result was defined as ‘best of three’, without any indeterminate results. Briefly, the test was performed using the Loopamp kit (Eiken Chemical Co. Ltd., Tokyo, Japan), using 7 µL of primers (FIP and BIP 40 pmol, Loop-F and Loop-B 20 pmol, F3 and B3 5 pmol), 12.5 µL reaction mixture (40 mM Tris-HCl, 20 mM KCl, 16 mM MgSO_4_, 20 mM [NH]_4_SO_4_, 0.2% Tween 20, 1.6 M betaine and deoxynucleotide triphosphates 2.8 mM each), 1 µL *Bst* DNA polymerase, 4 µL template DNA and distilled water to a total reaction volume of 25 µL. The reaction mixture was incubated in a realtime turbidometer (model LA 320CE with Loopamp software, Eiken Chemical Co. Ltd., Tokyo, Japan) at 65°C for 120 min. Positivity, defined as detection of exponential increase of turbidity was recorded at the first time point at which the change in turbidity increased by 0.1 optical density (OD) units/sec. Buffy coat derived DNA templates were used for the prospective evaluation of LAMP.

### ‘Scrub typhus infection criteria’ (STIC)

In order for the admission diagnosis to be sufficiently robust, a panel of reference criteria was defined which included parameters of high specificity, that define scrub typhus with a high level of confidence (Table 1).

**Table 1:** The scrub typhus infection criteria (STIC).

STIC	Detailed explanation
Isolation positive	In *vitro* isolation of *Orientia tsutsugamushi*
Admission IgM ≥1∶12,800	Single admission IgM titer of ≥1∶12,800
Dynamic serology	Paired serum samples with increasing IgM titers of minimum a four fold rise
≥2/3 PCR positive	Two out of three PCR based diagnostic assays are positive using 56 kD, 47 kD or *groEL* based target genes (i.e., minimum 2 different genes amplified)

The scrub typhus infection criteria (STIC) consist of diagnostic reference parameters of high specificity, aiming to define scrub typhus with a high level of confidence, irrespective of the timeliness of diagnosis. One or more of the following criteria have to be fulfilled for the diagnosis of ‘scrub typhus infection’. The STIC panel provides a robust diagnosis of scrub typhus, against which new diagnostic assays can be compared.

One or more of the following criteria had to be fulfilled for the diagnosis of ‘scrub typhus infection’: i. positive cell culture isolation of *O. tsutsugamushi*; ii. an admission IgM titer ≥1∶12,800; iii. a 4-fold rising IgM titer in paired serum samples; and iv. a positive result in at least two out of the three PCR assays described above.

While isolation and dynamic serology criteria are retrospective by nature, they provide the strongest evidence of active disease. Similarly the second-highest possible IgM titer measured upon admission (corresponds to ≥1∶12,800 in our laboratory) and two positive PCR results targeting two different genes provide high specificity and confidence of diagnosis. While single admission IgM titers in endemic areas are varied, and the various cut-offs used are notoriously unreliable, we opted to choose a ‘no-compromise’ titer for criterion to achieve maximal confidence in the diagnosis. The second-highest and highest measurable titers (1∶12,800 and 1∶25,600) cannot provide a 4-fold rise, hence a cut-off titer of 1∶12,800 was chosen as the highest single-titer for positivity upon admission.

### Data analysis

Diagnostic accuracy was calculated by comparing the LAMP assay results with a diagnostic reference comparator for each patient sample. Conventionally, scrub typhus infection is confirmed by *in vitro* isolation of *O. tsutsugamushi* or by a 4-fold rising IgM or IgG antibody titer in paired serum samples using the gold standard reference [3], [15]–[16]. In this study we used a combination of diagnostic criteria (serology, DNA and isolation) as the reference comparators, for simplification, these criteria were termed STIC (‘Scrub Typhus Infection Criteria’).

Analysis included cross-tabulation of reference comparators and LAMP assay results. Probability values for the comparison of positivity rates for LAMP with other assays were determined using McNemar’s test. Clinical and demographic results are reported as medians with interquartile range (IQR) and groups were compared using the Kruskal-Wallis test. Standard diagnostic accuracy indices were calculated using Stata/SE 10.0 (Stata Corp., College Station, Texas).

## Results

### Clinical and demographic characteristics

Of the prospectively recruited patients, 57/158 (36%) consisted of females and 99/158 (63%) were male. The median (IQR) age for females was 44 (34–55) and for males 39 (28–48). The median (IQR) fever days prior admission of all patients was 5 (3–7), and for patients fulfilling the STIC was 6 (4–7) days of fever. All recruited patients were in-patients and as such presented with symptoms at the more severe end of the disease spectrum – to date no reliable markers or panel of parameters to define disease severity has been evaluated for scrub typhus.

### Reference criteria (STIC) results

In this study of patients with ‘typhus-like illness’ 55/161 (34%) cases fulfilled the robust STIC reference criteria (Table 1). *In vitro* isolation was successful in 9/161 (6%), a ≥4-fold rise of IgM titer in paired sera was seen in 26/138 (19%), an admission IgM titer ≥1∶12,800 was present in 20/161 (12%) and two of the three PCR assays performed were positive in 27/161 (17%) of the patients (Table 2). The LAMP was assessed against these criteria, to determine sensitivity, specificity, NPV and PPV, as well as agreement to the STIC criteria (summarised in Table 2). The median number of ‘fever days prior to admission’ was 5 (IQR: 3–7) in the prospectively recruited patient group, data available for 156/161 (97%) of patients (Table 3).

**Table 2:** Overall and positivity percent agreement of LAMP results, when compared with other modalities.

Diagnostic Criteria		Positives	Diagnostic Accuracy of LAMP [95% CI]				McNemar
		n (%)	Sens (%)	Spec (%)	PPV (%)	NPV (%)	P-value
**Reference diagnosis**	STIC positive	55/161 (34)	53 [39–66]	94 [88–98]	83 [66–93]	79 [71–86]	**0.0004**
	Isolation positive	9/161 (6)	78 [40–97]	82 [75–87]	20 [8–37]	98 [94–100]	**<0.0001**
	Admission IgM ≥1∶12,800	20/161 (12)	70 [46–88]	85 [78–91]	40 [24–58]	95 [90–98]	**0.004**
	Dynamic serology	26/138 (19)	35 [17–56]	83 [75–90]	32 [16–52]	85 [76–91]	0.74
	≥2/3 PCR positive	27/161 (17)	82 [62–94]	90 [84–95]	63 [45–79]	96 [91–99]	0.06
**Admission diagnosis**	Realtime PCR (47kD)	28/161 (17)	79 [59–92]	90 [84–95]	63 [45–79]	95 [90–98]	0.11
	Realtime PCR (groEL)	35/161 (22)	77 [60–90]	94 [88–97]	77 [60–90]	94 [88–97]	1.0
	Nested PCR 56kD	23/161 (14)	83 [61–95]	88 [82–93]	54 [37–71]	97 [92–99]	**0.007**
	PanBio ICT IgM	31/159 (20)	61 [42–78]	88 [81–93]	54 [37–71]	90 [84–95]	0.5

The cohort consists of 161 clinical prospectively collected samples, follow-up sera were available for 138/161 (86%) samples. Probability values for the comparison of positivity rates for LAMP with the other assays were determined using McNemar's test: p-values in **bold** indicate that the proportion of LAMP positives and the proportion of the tested modality positives are significantly different, i.e. a low level of agreement.

**Table 3:** Dissection of discrepant LAMP assay results according to the ‘scrub typhus infection criteria’ (STIC).

STI Criteria	Reciprocal median IgM Titers		LAMP Result	(n = )	Percentage of positive results					Days of fever at sample collection
					PCR (% pos)			In vitro isolation	PanBio IgM ICT	
	Adm	Fup			groEL	47 kD	56 kD	(% pos)	(% pos)	(median, IQR)
≥4-fold dynamic IgM antibody rise	200	1,600	Positive	9	67	56	56	11	33	6 (4–7)
	50	400	Negative	17	18	6	6	0	12	5 (3–7)
Admission IgM antibody titer ≥1∶12,800	25,600	25,600	Positive	14	100	93	86	29	86	7 (5–8)
	25,600	25,600	Negative	6	17	17	17	17	100	8.5 (5–10)
≥2/3 PCR assays positive	12,800	25,600	Positive	22	100	100	86	23	77	7 (4–8)
	100	25,600	Negative	5	100	80	60	20	60	7 (6–7)
In vitro isolation positive	25,600	25,600	Positive	7	86	71	71	100	57	5 (3–8)
	12,800	12,800	Negative	2	50	50	50	100	50	4.5 (4–5)

The LAMP results of each individual sub-group of STIC were grouped into positive/negative to allow for comparisons to other diagnostic modalities. Serological median reciprocal admission (Adm) and follow-up (Fup) IgM titers are shown as well as supportive diagnostic evidence from PCR, isolation and rapidtests (percentage of positive results). Discrepancies had no association with ‘days of fever / illness’ prior to admission.

The standard IgG-based IFA used in non-endemic areas, was performed in addition to the IgM-based IFA, but the results not used diagnostically in this manuscript as the patient samples are derived from a highly endemic area. High endemicity, presumably, resulted in a high number of patients with ‘diagnostically high’ admission IgG levels (n = 61/161) based on a 1∶400 cutoff titer [16], as compared with those with high IgM levels (N = 42/161). The number of dynamic rises though was similar for both IgG and IgM (40/161 and 43/161 respectively). For this reason for this study in this setting we used only IgM IFA results.

### Diagnostic accuracy of LAMP

#### The acute setting

The currently most practical diagnostic assays for use in the setting of acute illness (‘point-of-care’) are ICT rapidtests and, with more limitations, realtime PCR assays. LAMP was assessed against these assays in this analysis. LAMP and *groEL* realtime PCR assays detected 35/161 (22%) samples, followed by the PanBio ICT 31/161 (19%) and the 47 kDa realtime PCR assay 28/161 (17%). Using the STIC criteria as reference comparator, the LAMP assay demonstrated high diagnostic specificity (95% CI) of 94% (88–98) and a sensitivity of 53% (39–66), and the IgM-based rapidtest PanBio ICT performed with a sensitivity of 47% (34–61) and a specificity of 95% (89–98). When the LAMP and PanBio ICT IgM rapid test were combined, an increased sensitivity of 67% (53–79) was achieved with a specificity of 91% (83–95). Comparisons of the *groEL*, 47 kD and 56 kD PCR were not performed as they are constituents of the STIC criteria.

#### Dissection of discrepant results

To understand the relative (dis)advantages of a molecular test when compared against the broad diagnostic panel, the discrepancies in positivity agreement between the assays were dissected at the individual diagnosis level (Table 3).

Discordant results with STIC criteria: In the ‘admission IgM ≥1∶12,800’ group, the number of LAMP negative cases was 6/20 (30%). Despite high admission titers, these cases had low support from the other PCR assays (17% positivity for all PCRs) and in the ‘≥2/3 PCR positive’ group there were 5/27 (19%) LAMP negative cases, which had strong support through very high positivity rates in the other PCR modalities. In the ‘isolation positive’ group 2/9 (22%) LAMP negative cases were found, one of which was realtime PCR positive. The largest proportion of STIC sub-criterion positive results that were discrepant to LAMP results was found in the ‘dynamic serology’ group, which included 17/26 (65%) cases. However these LAMP negative cases demonstrated low serological and low PCR support by the other modalities (Table 3).

### The effect of sample timing on PCR and IgM antibody based assays

**Figure 1:**
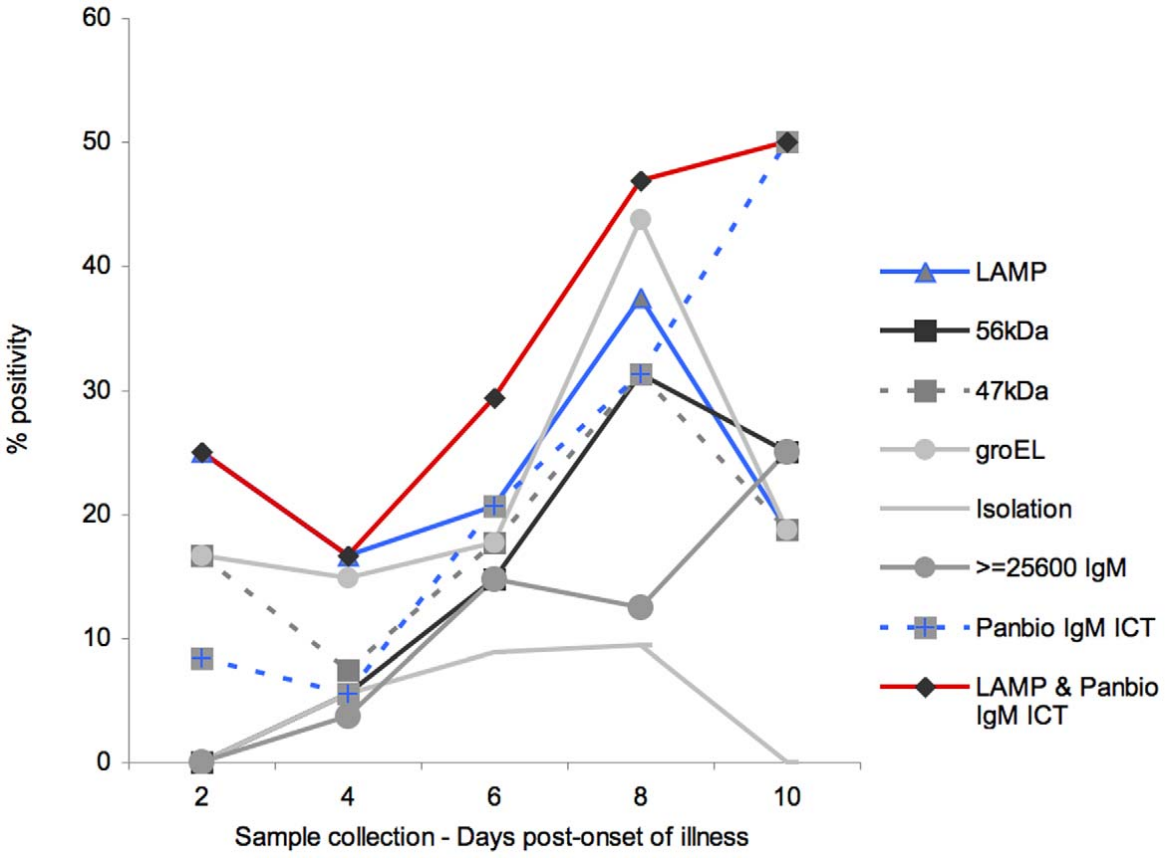
Effect of sample timing on admision diagnostic positivity rates. Sample timing in terms of ‘days of fever prior diagnosis’ was significantly associated with LAMP positivity rates. Overall, the LAMP assay was superior at demonstrating positivity up 8 days of fever prior admission. Combination of the LAMP assay and the PanBio IgM ICT rapid test improved the overall sensitivity and expanded the temporal spectrum of *O. tsutsugamushi* detection in the acute setting.

At 2 days of fever the admission assays were performed as follows: LAMP assay had a positivity rate of 25%, higher than that of the *groEL* and 47 kD realtime assays (both 17%), and the antibody-based rapidtest PanBio ICT was lowest with 8% (Figure 1).

The PCR based assays peaked their performances at day 8 of fever, with positive detection rates of 38% for LAMP, 44% for the *groEL* realtime PCR assay and 31% for the 47 kD and 56 kD assays. These rates were in temporal harmony with the isolation positivity rates, which ranged from 4 to 8 days of fever. However, all DNA-based assays (including isolation) markedly dropped to approximately 20% at 10 ‘days of fever’. The PanBio ICT assay, after low positivity rates in the early phase of illness, demonstrated a steady increase of sensitivity and surpassed the diagnostic efficacy of DNA based assays at day 10 with a 50% positivity rate (Figure 1).

Combining the LAMP and PanBio ICT assays increased the overall proportion of positivity across all days of sample collection studied, ranging from 25% at day 2 to 50% at day 10 post onset of illness (Figure 1). The difference in positivity agreement between the LAMP assay and the PanBio IgM ICT antibody-based assay against the STIC criteria was statistically significant until day 8 (all p values<0.03, data not shown), reflecting the superiority of LAMP in the early disease course. The combination of the LAMP assay with the PanBio IgM ICT results demonstrated a significantly higher positivity agreement with the STIC criteria (p = 0.01) at day 8 and onwards compared to the LAMP assay alone.

## Discussion

This study aimed to assess the true diagnostic utility of LAMP in a clinical setting, by evaluating its diagnostic accuracy, in absolute terms, when compared to gold standard established (retrospective) diagnostic criteria in the acute setting, but also in relative terms, compared to available diagnostic assays used upon admission, thus supporting clinical management. This form of evaluation was challenging for LAMP, as it was based on a prospective cohort in an endemic area using broad robust diagnostic criteria across different methodologies.

The increased use of molecular diagnostic methods in the diagnosis of scrub typhus, has highlighted the difficulties of comparing DNA-based with antibody-based assays [14]. Diagnostic methods in the acute setting require more attention with regards to the wide antigenic heterogeneity of *O. tsutsugamushi* [17] and the currently suboptimal and retrospective gold standard [3]. Comparison of a DNA-based method to a serology-based method would limit the identification of true benefits a novel methodology could accompany. For these reasons, we have created a stringent and inclusive set of criteria for the definition of scrub typhus infection (STIC) using serological, gene-based and *in vitro* isolation methods to determine a broad final diagnosis with high specificity. The perfect diagnostic tool for the diagnosis of scrub typhus would have to cover the same (or an even greater) patient spectrum in a single assay, as the STIC criteria do comprising multiple assays.

Unfortunately the absolute sensitivities of all individual assays that are currently useful in the acute setting (i.e. realtime PCR and ICT tests) were lower or equal to 56% for the diagnosis of scrub typhus, when compared to the retrospective and definitive STIC criteria (based on positive and negative results, data not shown). The low sensitivities for these individual assays reflect the current inability of a single assay to cover the spectrum of diagnostic positivity for all ligands included in the STIC criteria. On the other hand, it underlines the usefulness of these criteria for evaluation of different diagnostic methods, and also demonstrates how severely under-diagnosed this disease is, as almost every second case may go undiagnosed upon admission, even if the currently most sensitive methods are applied.

On dissection of the STIC criteria by subgroup analysis, major discrepancies between the LAMP assay and the criterion ‘dynamic serology’ were revealed. The highest level of disparity occurred when admission sample IgM antibody titers determined by IFA were low (i.e. median titers of 1∶50 rising to 1∶400). Only a very small proportion of patients with a dynamic rise in IgM antibody titer that exhibited low IgM antibody titers had additional supportive evidence of acute scrub typhus (Table 3). Alternatively, if these cases with low-rising IgM titers are not scrub typhus, potential cross-reactivity of antibodies generated from other (endemic) infections must be considered, for example murine typhus or leptospirosis or co-stimulation of the humoral immune response in a primed population with high background titers by another infection.

The intended ideal role for the LAMP assay is in the acute point-of-care (POC) setting where there is limited availability of sophisticated molecular diagnostics. Comparison of the relative positivity rates of LAMP and the easy-to-use PanBio ICT against the STIC criteria revealed slightly better sensitivity for the LAMP assay 53% vs. 47% for the ICT, but similar specificities of 96%. While the overall agreement of positive and negative results for both assays was 82% (131/159), the agreement of positive results was low at 68% (19/28). This finding could be explained in a dynamic context as in the early stages of infection high LAMP positivity paralleled low PanBio ICT positivity rates (Figure 1). After 10 ‘days of fever’, the LAMP positivity rates decreased (possibly reflecting a decrease of haematogenous dissemination of *O. tsutsugamushi in vivo*) but patient IgM levels continue to rise, allowing the PanBio ICT to outperform the LAMP assay at this timepoint. This finding supports the potential benefit of combining the LAMP [Sensitivity  = 53% (39–66), Specificity  = 94 (88–98)] and PanBio IgM ICT [Sensitivity = 47% (34–61), Specificity = 95% (89–98)] as the two most practical POC assays; in an ‘OR’ Boolean operator manner. The benefit of combining these tests is reflected by an increase of the overall diagnostic sensitivity to 67% (95% CI: 53–79), when compared to STIC criteria. With a total of 47/161 (29%) patients diagnosed upon admission and a specificity of 91% (95% CI: 83–95) this modality demonstrated the best option for diagnostic use in the acute setting and approached the diagnostic accuracy of the retrospective STIC reference criteria.

Further, the combination of these two assays also expanded the temporal spectrum of diagnosis in the acute setting, as it utilises DNA and/or antibody positivity endpoints. This approach has the potential for future improvement if advances in antibody-based ICT technology and simplification of DNA extraction methods are made available in the future. As an analogy, the combination of Dengue NS1 antigen and IgM antibody assays for the diagnosis of acute dengue infections has dramatically increased the sensitivity of admission diagnosis [18]–[19].

It is notable, that the median number of ‘fever days’ prior to admission for all patients from N-Thailand was 5 with an IQR of 3–7 and this diagnostic window is sufficiently covered by the LAMP assay.

In conclusion, in a clinical evaluation against a broad and robust panel of diagnostic criteria the LAMP assay performance was similar to that of the realtime and conventional nested PCR assays. The diagnostic discrepancies between PCR assays and ‘dynamic serology’ were associated with low admission IgM antibody titers (i.e. low antibody titers and PCR negativity) and warrant further investigation into endemic background antibody levels, which should include consideration of potential immune cross-reactivity or non-specific polyclonal activation due to other diseases.

The LAMP methodology was unsurprisingly unable to cover the whole spectrum of diagnostic capabilities as defined by our STIC criteria. However the combination of a DNA- and antibody-based detection method increased sensitivity with minimal reduction of specificity, and expanded the timeframe of adequate diagnostic coverage throughout the acute POC phase of scrub typhus. The LAMP assay is simple and inexpensive, performs like a realtime PCR and can be considered a valid molecular method for the early diagnosis of scrub typhus.
